# Association between temporomandibular disorders and anxiety: A systematic review

**DOI:** 10.3389/fpsyt.2022.990430

**Published:** 2022-10-13

**Authors:** Erick Alves dos Santos, Beatriz Rodrigues Risuenho Peinado, Deborah Ribeiro Frazão, Yago Gecy de Sousa Né, Nathalia Carolina Fernandes Fagundes, Marcela Baraúna Magno, Lucianne Cople Maia, Rafael Rodrigues Lima, Renata Duarte de Souza-Rodrigues

**Affiliations:** ^1^Laboratory of Functional and Structural Biology, Institute of Biological Sciences, Federal University of Pará, Belém, Pará, Brazil; ^2^School of Dentistry, Faculty of Medicine and Dentistry, University of Alberta, Edmonton, AB, Canada; ^3^Department of Pediatric Dentistry and Orthodontics, School of Dentistry, Federal University of Rio de Janeiro, Rio de Janeiro, Brazil

**Keywords:** Temporomandibular Joint Dysfunction, anxiety, psychosocial impairment, systematic review, oral health

## Abstract

Temporomandibular Joint Dysfunction (TMD) is an umbrella term that includes musculoskeletal and neuromuscular conditions affecting the temporomandibular joint. The present systematic review aimed to verify whether there is a specific association between TMD and anxiety. The searches were carried out in electronic databases, including PubMed, Scopus, Web of Science, and LILACS, without restrictions on publication date and language. The acronym PECO was used, whose participants (P) were humans exposed to TMD (E), compared to participants without TMD (C) and the presence of anxiety as an outcome (O). After the search retrieval, the duplicates were removed, and the articles were evaluated by title and abstract, following our inclusion and exclusion criteria; then, the papers were read and thoroughly assessed. After selection, the methodological quality was performed using the Newcastle-Ottawa Scale (NOS) for observational studies. The Grading of Recommendations, Assessment, Development, and Evaluation (GRADE) tool was used to assess the level of evidence. A total of 710 studies were found, and 33 articles were considered eligible and were included for the qualitative synthesis and the level of evidence assessment. The studies confirmed the association between anxiety and DTM, although there was a low certainty of evidence among the selected studies. Most articles showed a low risk of bias. Although the limitations of this systematic review, it suggested a significant association between anxiety and TMD, as well as highlights possible directions for future research.

## Introduction

For the American Academy of Orofacial Pain (AAOP), Temporomandibular Joint Dysfunction (TMD) is described as an umbrella term that includes musculoskeletal and neuromuscular conditions, which can affect the Temporomandibular Joint (TMJ), masticatory muscles, and/or their associated structures (1). Usually, TMD is classified into subgroups, articular or muscular origin. In the first case, signs and symptoms are related to TMJ. And in the second case, symptoms and signs are related to stomatognathic structure (2).

The etiology of TMD is not fully understood; however, it is known to has a multifactorial origin, which can result from abnormal interference from psychological, physiological, structural (occlusion and trauma), and postural (parafunctional habits) and genetic conditions. These conditions may compromise the homeostasis state of the stomatognathic system, since they can act as initiating, predisposing, and perpetuating factors, resulting in the appearance of TMD signs and symptoms (3–5).

A recent study investigated the prevalence of temporomandibular disorders among the general population and concluded that the overall prevalence of TMD was approximately 31% on adults/elderly and 11% for children/adolescents. When evaluated according to joint or muscle subgroups, the authors found that the most prevalent TMD was disc displacement with reduction (DDwR), that was present in approximately 26% in adults/elderly and 7.5% in children/adolescents (2). The literature presents a range of acute or chronic symptoms commonly reported, such as pain and/or discomfort in the TMJ, ears, chewing muscles, eyes, and face; psychological distress; physical disability; noises, crackles or clicks on the joint; locking or considerable limitation of jaw opening, closing and laterality movements (3, 6, 7).

Previous studies have shown that psychosocial disorders and psychosocial impairment play an important role in the development of TMD (5, 8–14). The Orofacial Pain Prospective Evaluation and Risk Assessment (OPPERA) highlights that the prevalence of psychosocial factors is higher in TMD patients compared to the healthy individuals (15). It is presumed to act as both initiating and perpetuating factors (11). Anxiety stands out as a comorbidity frequently associated with TMJ disorders, as it can change pain sensations and release neurotransmitters related to parafunctional habits. Also, anxiety can potentiate the hyperactivity of chewing muscles associated with TMJ, resulting in joint overload (4, 5, 7, 16). It can be classified according to the frequency of manifestations: state anxiety, that is a pathological emotional response of varying intensity on a particular stressor, specific, unique; and trate anxiety, an emotional state changed variable intensity trend of reactions to different stressors, treated as a stable behavioral characteristic (17).

Recently, a systematic review (18) investigated whether there is a subtype of temporomandibular disorder that is more associated with the occurrence and severity of both anxiety and depression (together in the same patient) and the authors concluded that patients with myofascial pain are more anxious and depressed than others. In this way, it is clear how necessary to investigate separately biopsychosocial factors during the evaluation of TMD patients (5, 14). Thus, based on previously reported findings on the relationship between biopsychosocial factors and TMD, the present systematic review aims to investigate whether there is association between anxiety and TMD, regardless of the subtype.

## Methodology

### Protocol and registration

This systematic review was designed in accordance with the Preferred Reporting of Systematic Review and Meta-analyses (PRISMA) (19) and registered with Open Science Framework under the doi: https://doi.org/1017605/OSF.IO/YN3VJ.

### Eligibility criteria and search strategy

The research question of this systematic review was “Is there an association between temporomandibular disorders and anxiety?” Therefore, our eligibility criteria were based on the PECO acrostic to look for observational studies in adult humans (P), exposed to a diagnostic of TMD of any kind, such as muscular, articular, or pain-related (TMD) (E), compared to participants without TMD diagnostic (C), having as an outcome the presence of anxiety (O). There was no restriction of year of publication nor language. Our exclusion criteria were case reports, descriptive, opinion, technical, animal, and *in vitro* studies.

The searches started in January 2022, with no language defined for the results, in which 4 (four) digital databases were consulted: PubMed, Scopus, Web of Science, and LILACS. It was planned in the registered protocol to search 8 databases but only 4 could be covered, excluding the grey literature. Besides, after selecting the articles, a hand research was done through the final studies' references. All publications complied with pre-defined combination requirements based on Medical Subjects Headings (MeSH) and entry terms. Boolean Operators (AND, OR) were used with different MeSH terms and related.

### Selection process

The selection process was carried out by two examiners (EAS and BRRP), and a third examiner (DRF) was consulted to reduce the chances of errors and disagreements between the two evaluators. All the references collected were managed using EndNote Software, VX7. Duplicate studies were considered only once after identification and exclusion. After the exclusion of duplicate studies, the bibliographies were again submitted to inclusion and exclusion criteria based on the title and abstract analysis. The resulting articles were read in full and excluded if they did not comply with the authors' PECO search strategy.

### Data extraction

An extraction table was made by two authors (EAS and DRF), separating the data in various aspects: author and year of publication, study design, country of study, characteristics of the participants (exposed and control group), sample size and age group, TMD and anxiety assessment methods, statistical tests and results obtained.

### Methodological quality

The Newcastle-Ottawa Scale (NOS) assessed the methodological quality of the selected studies. According to the NOS' guideline, the system of stars/asterisks (^*^) was adopted to indicated the score attributed to each article in each domain of the NOS after two authors had performed the evaluation and judgement. For case–control and cohort studies, the maximum number was nine stars distributed in three domains: Selection (adequate definition of cases, representativeness of cases, selection of controls, and definition of controls), comparability, and exposure (exposure verification, the only evaluation method for cases and controls and non-response rate). For cross-sectional studies, the total number of stars could vary from 0 to 10, in which they were also distributed in three domains: selection (sample representativeness, sample size, non-response rate, and exposure verification), comparability, and outcome (outcome assessment and statistical test used). The criteria for each domain are exposed in Supplementary Table 1.

### Level of evidence

GRADE (Grading of Recommendations Assessment, Development and Evaluation) was used to analyze the quality of evidence. GRADE is a grading system for the quality of evidence and strength of health recommendations. When serious or extremely serious issues related to the risk of bias, inconsistency, indirectness, imprecision, and publication bias are observed, the certainty of evidence decreases by one or two. If the effect of all plausible confounding factors is minimized or suggests a spurious effect, the quality of evidence tends to increase. The magnitude of an effect and dose-response could not be evaluated in the present GRADE and was not considered. GRADE was sub-grouped according to anxiety.

## Results

### Studies selection and characteristics

**Figure 1:**
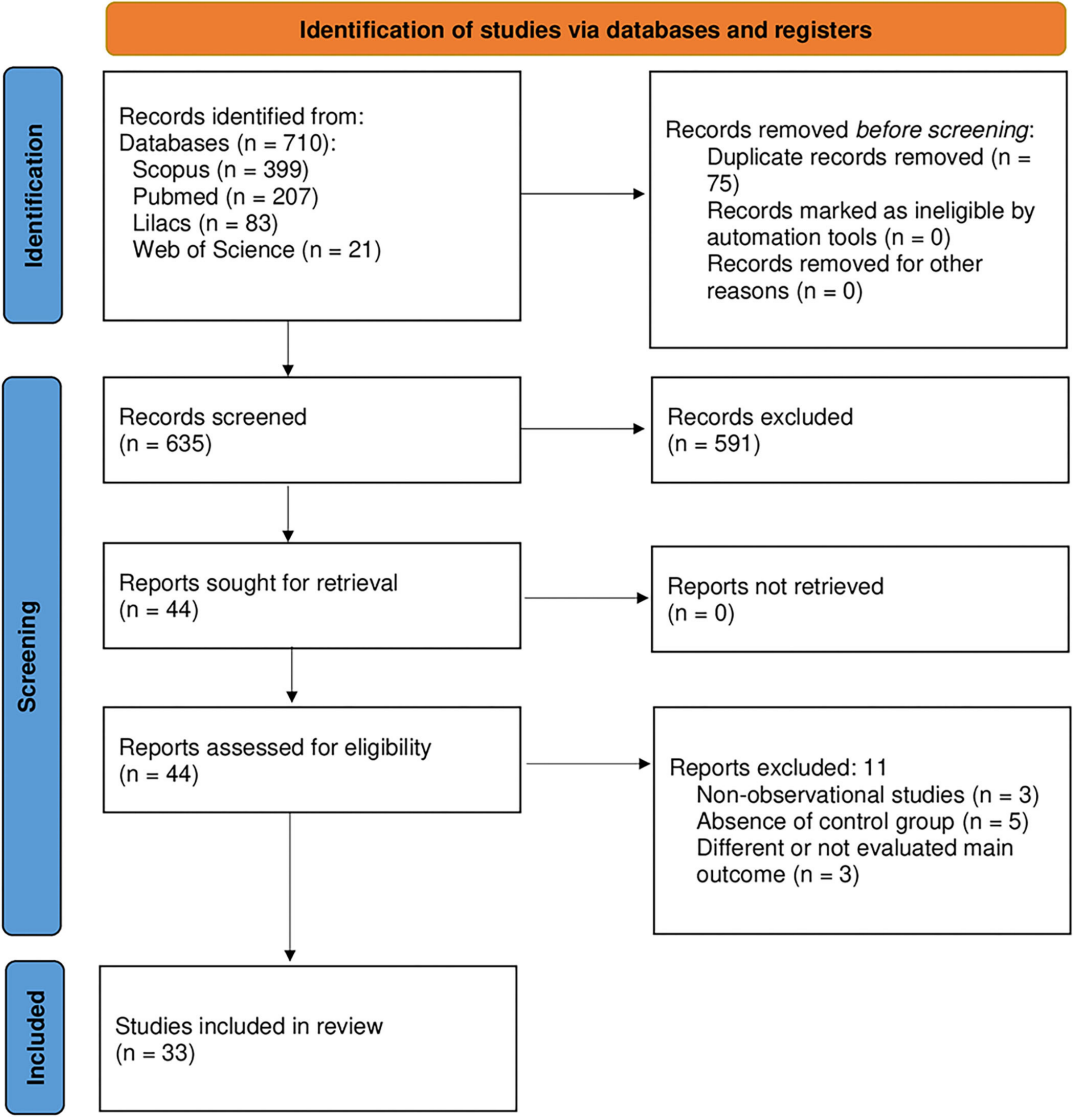
Flow diagram of the selected studies according to the PRISMA protocol.

In total, 710 manuscripts were found through electronic database searches, 75 of which were rejected due to duplication criteria. The titles and abstracts of 635 studies were examined, resulting in the exclusion of 591 articles and the selection of 44 to read the full text. All articles that did not apply to the PECO established by the authors were removed. The reasons for exclusion were as follows: non-observational studies (20–22), the absence of a control group (23–27). Therefore, this systematic review included 33 studies for qualitative analysis (Figure 1).

### Results of individual studies

There were 13 cross-sectional studies and 20 case–control studies among the 33 studies included. The research question was answered after analyzing the data extraction table (Table 1), in which 30 studies indicated an association between the presence of TMD and anxiety, while only four articles did not indicate such an association in their results. Some studies have developed more detailed indexes for anxiety assessment, presenting, in addition to the diagnosis of this pathology, the classification into two major groups: trait anxiety and state anxiety.

**Table 1:** Extraction of the studies' results.

**Author/ Year/ Country/ Study design**	**Participants**			**TMJ Disorders evaluation**	**Anxiety evaluation**	**Statistical analysis**	**Results**
	**Source of sample**	**Sample size**	**Age (years)**				
Solberg et al. (28) United States of America Case-control	University of Minnesota School of Dentristy	TMJ Disorders: 29 Controls: 29	Mean age for TMJ disorders and controls: 14–63 years	Histories, regional examinations, dental and lateral transcranial oblique radiographs for all patients	Minnesota Multiphasic Personality Inventory (MMPI)	*t*-test *p < * 0.001	Half of the symptom group showed clinical signs of greater anxiety than the matched control group as measured by the MMPI. The control group was essentially free of elevated levels of anxiety.
Moss and Adams (29) United States of America Case-control	Subjects were recruited from the community through radio advertisements and as referrals from local dentists	TMJ Disorders: 20 Controls: 10	Mean age for TMJ Disorders: 28.7 (19–41) years Mean age for Controls: 25.8 (22–32) years	TMJ pain and TMJ sound of click	Spielberger State-Trait Anxiety Inventory (STAI)	One-way ANOVA *p < * 0.05 *post hoc*	The results of the STAI indicated that there were no differences among groups in relation to state anxiety.
Southwell et al. (30) Scotland Case-control	Prosthetics Department at the Edinburgh Dental Hospital	TMJ Disorders: 32 Controls: 32	Mean age for TMJ Disorders: 36 ± 15.8 years Mean age for Controls: 36.2 ± 19 years	Classic triad of symptoms.	Eysenck Personality Questionnaire (EPQ); Spielberger State Trait Anxiety Inventory (STAI); Pennybaker Inventory of Limbic Languidness (PILL).	*t*-test *p < * 0.005	TMJ patients showed higher state and trait anxiety levels on the Spielberger scales, but only the trait difference was significant (*P < * 0–0.05). When females were compared, the only significant difference between TMJ patients and controls was in trait anxiety (TMJ mean - 43-2, control mea*n =* 34-8; t = 2-80, *P < * 0–0.05)
Schroeder et al. (31) Germany Case-control	Department of the Clinic of Dental Surgery (Charité) and Institute of Physiology	TMJ Disorders: 30 Controls: 25	Range for TMJ disorders and controls: 19–66 years	Dysfunction Index proposed (32); orthopantomographic examination to assess the structure of the TMJ; examination of their occlusion and electromyographical investigation.	Scale ranging from 1–5 (from very relaxed to extremely tense) after the electromyographical investigation and the Hospital Anxiety and Depression Scale questions (HADS) (33).	Kruskal-Wallis-Test *P < * 0.05	Anxiety developed as conditioned reactions to pain. In agreement with this interpretation, patients with high anxiety values more often reported their mood during the experiment as tensed or stressed than did those with low anxiety values.
Zarh et al. (34) United States of America Case-control	TMJ clinic at University Hospital and Clinic, Iowa City, Iowa College of Dentistry, University of Iowa, Iowa City, Iowa.	TMJ Disorders: 98 Controls: 98	Range for TMJ disorders and controls: 18–65 years	Clinical examination (tenderness of muscles of mastication on palpation, tenderness of TMJ on lateral palpation, audible sounds in the TMJ on opening and closing, etc.)	The Crown Crisp Experimental Index (CCEI)	The paired Student *t* test	TMD patient in this study is more anxious about bodily concerns than the non TMD subject Stockstill and Callahan (35) United States of America Case-control	From several dental care facilities in the Lincoln, Nebraska, area, including a dental college clinic and several private practitioners.	TMJ Disorders: 47 Controls: 49	Mean age for TMJ Disorders: 33.8 ± 9.95 Mean age for Controls: 32.9 ± 10.2	Patients who had been in treatment for 1 year or less (Clinical history)	Taylor Manifest Anxiety Scale	Student's *t*-test Mann-Whitney *U* test *P < * 0.05	No significant differences were found between the subsamples (anxiety: i[58] = 0.687, *P* = NS)
Curran et al. (36) United States of America Case-control	Orofacial Pain Center at the University of Kentucky, College of Dentistry and university population (including introductory psychology courses for researcb credit)	TMJ Disorders: 23 Controls: 23	Mean age for TMJ Disorders: 26.9 years Mean age for Controls: 27.4 years	Research Diagnostic Criteria for Temporomandibular Disorders (RDC);	State-Trait Personality Inventory (STPI); Emotion Assessment Scale (EAS);	*t*-tests *p < * 0.05	The patients with TMD also indicated greater anxiety based on the trait measure of the STPI than did the control subjects (TMD X = 23.83, MC X = 19.35) [t/45] = 2.40, *P < * 0.03). The patients with TMD indicated greater levels of anxiety X = 24.70| relative to control subjects X = n.87) (¡[45] = 2.55, *P < * 0.02) on the EA
Jones et al. (37) Canada Case-control	Department of Dentistry at Victoria Hospital, London, Ontario, Canada;	TMJ Disorders: 36 Controls: 39	Mean age for TMJ Disorders: 31.86 ± 11.40 Mean age for Controls: 22.28 ± 6.37	Research Diagnostic Criteria for Temporomandibular Disorders (RDC); The Temporomandibular Joint Pain and Dysfunction Index (TMJPDI)	Positive and Negative Affect Scale (PANAS); Visual Analog Scale (VAS) on 15 cm; The Symptom Checklist 90–Revised (SCL-90R);	*t*-test ANOVA MANOVA SPSS/PC+ *p* = 0.05	The TMD group showed significant negative relationships between cortisol response and self-reported symptoms of anxiety. The TMD group showed greatly increased salivary cortisol concentrations to almost 12 nmol/l in response to the stress protocol.
Sirirungrojying et al. (38) Case-control Thailand	Dental Hospital, Faculty of Dentistry, Prince of Songkhla University	TMJ Disorders: 62 Controls: 67	Mean age for TMJ Disorders: 31.5 ± 10.9 years Mean age for Controls: 33.5 ± 10.2 years	Criteria of the American Academy of Craniomandibular Disorders	SCL-90 Questionnaire	*t*-test *p < *0.05	Significant difference between TMD patients and dental patients at *P < * 0·05. Thus, the TMD patients in the study were more depressed and anxious than the other group.
Velly et al. (39) Canada Case-control	Jewish General Hospital (JGH) and the Montreal General Hospital (MGH), Montreal, Quebec, Canada.	TMJ Disorders: 83 Controls: 100	Mean age for TMJ disorders and controls: 18–60 years	Extra-oral and intra-oral clinical examinations; Research Diagnostic Criteria for Temporomandibular disorders (RDC/TMD)	The symptom check list 90 revised questionnaire (SCL-90R)	ratio test *p* = < 0.05	A mid-level of phobic anxiety appeared to be related to chronic pain. Higher levels of anxiety, but not depression, were associated with chronic miofacial pain. Manfredini et al. (40) Italy Case-control	Section of Prosthetic Dentistry, Department of Neuroscience, University of Pisa, Italy	TMJ Disorders: 87 Controls: 44	Mean age for TMJ disorders and controls: 25.8 (19–66) years	Research Diagnostic Criteria for Temporomandibular disorders (RDC/TMD) axis-I categories	Self-report questionnaire PAS-SR (version of SCIPAS).	One-way ANOVA *Post hoc* test *P < * 0.05	Myofascial pain subjects presented significantly higher scores PAS-SR domains investigating of anxiety. Myofascial pain patients showed the highest prevalence of anxiety psychopathology when compared with TMD-free.
Pallegama et al. (41) Democratic Socialist Republic of Sri Lanka Case-control	Oral Medicine Clinic at the Dental Hospital of the University of Peradeniya, Sri Lanka.	TMJ Disorders:38 Controls: 41	Mean age for TMJ Disorders: 29 ± 10.3 Mean age for Controls: 27.3 ± 8.2	Protocol adopted from the one used by Liu et al.	The self-evaluative, Spielberger's state and trait anxiety inventory (STAI)	Chi-square test Hapiro–Wilk test Levene's test Box's M-test Bonferroni *post-hoc* test *P < * 0.05	Multiple comparisons among the three groups revealed that the patients with TMD exhibited significantly higher levels of trait anxiety than controls (*P < * 0.01) Muscle related TMD patients all together showed significantly raised trait anxiety levels and neuroticism scores compared with healthy individuals.
Saheeb and Otakpor (42) Nigeria Case-control	Department of Oral and Maxillofacial Surgery at the University Benin Teaching and Hospital Benin City, Nigeria.	TMJ Disorders: 24 Controls: 24	Mean age for TMJ Disorders: 43.9 ± 15.9 Mean age for Controls: 44.1	Research Diagnostic Criteria for Temporomandibular disorders (RDC/TMD)	State-Trait-Anxiety Inventory (STAI); Hospital Anxiety and Depression Scale (HADS); The 28- item General Health Questionnaire (GHQ-28).	Chi-square test (x^2^) *P < * 0.05	This study identified a significantly higher prevalence rate of psychiatric among the temporomandibular joint pain and dysfunction patients compared with a control group. In 25% generalized anxiety and 12.5% dysthymia as the main comorbid psychiatric diagnoses in these patients.
Xu et al. (43) China Cross-sectional	Temporomandibular Joint Treatment and Joint and Oral and Maxillofacial Pain Center, School of Stomatology, Peking University	TMJ Disorders: 338 Controls: 1,338	Mean age for TMJ disorders and controls: 29.5 ± 10.5 (18–65) years	Patients in TMD treatment	SCL-90 Questionnaire	t-test ANOVA *P < * 0.05	The anxiety score of TMD patients was higher than the control population. This difference is statistically significant (*p < * 0.05)
Fernandes et al. (44) Brazil Cross-sectional	Undergraduates in Dentistry at University of Brasilia	TMJ Disorders: 225 Controls: 75	Mean age for TMJ disorders and controls: 17–25 years	Auto-applicable questionnaires Fonseca's Index	Spielberger's Trait–State Anxiety Inventory	Kruskal-Wallis and Pearson correlation *p < * 0.001	Pearson's Correlation Test, allowed to verify that the degree of TMD showed a positive correlation (r= 0.1872; *p* < 0.01) for both trait-anxiety ad state, that is
							increase in TMD is directly proportional to the increase in the level of anxiety
Vedolin et al. (45) Brazil Case-control	Bauru School of Dentistry (University of São Paulo, Brazil).	TMJ Disorders: 29 Controls: 16	Mean age for TMJ Disorders: 20 years Mean age for Controls: 19.5 years	Research Diagnostic Criteria for Temporomandibular disorders (RDC/TMD)	Beck Anxiety Inventory (BAI) (derived from the Cornell Medical Index)	Tukey's test Mann–Whitney test Friedman's test Chi-squared test	There was no difference between groups in anxiety and stress at any time (*P* > 0.05). When comparing the levels of anxiety and stress between times in each group, T2 had higher values, although not statistically significant for both groups.
Giannakopoulos et al. (8) Germany Case-control	Department of Prosthodontics of the University Hospital of Heidelberg	TMJ Disorders:131 Controls: 91	Mean age for TMJ disorders and controls: 42 ± 15.4	Research Diagnostic Criteria or Temporomandibular disorders (RDC/TMD)	Hospital Anxiety and Depression Scale (HADS)	One-way ANOVA *t*-tests	Using the HADS scale to measure anxiety, this study showed that anxiety does not seem to be significant for chronic TMD patients.
Lajnert et al. (46) Croatia Case-control	Dental Polyclinic, School of Medicine in Rijeka, and Clinic for Psychotrauma of the Rijeka University Hospital Center	TMJ Disorders: 60 Controls: 30	Mean age for TMJ disorders and controls: 38.5 ± 12 (22–67) years	Research Diagnostic Criteria for Temporomandibular disorders (RDC/TMD)	Emotions Profile Index Life Events Scale	Kolmogorov-Smirnov test. One way ANOVA with Scheffe *post hoc* (*p* = 0.05). Pearson's coefficient of correlation (r).	The acute patients self-perceive higher levels of anxiety in relation to the control group; Acute pain is often coupled with anxiety. Patients suffering from the TMD's exhibit higher levels of anxiety compared to the healthy ones.
Monteiro et al. (47) Brazil Cross-sectional	São Paulo State University, Sagrado Coracão University and Thathi COC University	TMJ Disorders:49 Controls: 101	Mean age for TMJ disorders and controls: 17–30 years	Research Diagnostic Criteria for Temporomandibular Disorders (RDC/TMD)	Spielberger's Trait–State Anxiety Inventory (STAI-T and S).	Chi-square test *p < * 0.05	The correlation between trait-anxiety levels and chronic orofacial pain degrees was significant and positive (*p* = 0.0154; *p < *0.05).
Bezerra et al. (48) Brazil Cross-sectional	Center of Biological and Health Sciences (CCBS), University of Paraíba (UEPB), in Campina Grande	TMJ Disorders: 210 Controls: 126	Mean age for TMJ disorders and controls: 18–38 years	DMF Anamnestic Index	Anamnesis Index and the State-Trait Anxiety Inventory (IDATE).	Pearson's Chi-square Fisher Exact tests Confidence interval 95%	It was observed a higher prevalence of anxiety of moderate/high level for TMD individuals and of low level for TMD free individual.
Boscato et al. (49) Brazil Cross-sectional	Individuals in the Midwest region of Santa Catarina, a developed state of Brazil	TMJ Disorders: 247 Controls: 321	Mean age for TMJ disorders and controls: 35–74 years	Clinical examination	Hospital Anxiety and Depression Scale (HADS) (Seven itens for anxiety (HADSa)	Chi-squared test Poisson regression *P* ≤ 0.05	TMD occurrence increased with the anxiety level (*P* = 0.001). Women had a higher risk of presenting TMD, as well as individuals with mild and high levels of anxiety. that there is an increased risk of TMD in women and
							individuals with higher levels of anxiety. Strong association between TMD and psychological factors as higher levels of anxiety resulted in moderate and severe TMD with statistically significant difference.
Smriti et al. (50) India Cross-sectional	VSPM's Dental College	TMJ Disorders: 28 Controls: 122	Mean age for TMJ disorders and controls: 18–25 years	Self-administered anamnestic questionnaire (modified version of Helkimo's anamnestic index)	Zung self-rating scale	Chi-square	This study has a statistically significant association between TMD degree and anxiety.
Lemos et al. (51) Brazil Cross-sectional	Academics of undergraduate course in Dentistry of a public university in northeastern Brazil	TMJ Disorders: 50 Controls: 85	Mean age for TMJ disorders and controls: 18–25 years	Adapted anamnestic questionnaire; Clinical examination	Hospital Anxiety and Depression Scale (HADS)	Chi-square Fisher's exact test *p < * 0,05	In the sample evaluated, anxiety was associated with jaw locking (*p =* 0.031), fatigue during chewing (*p =* 0.025) and difficulty moving the jaw (*p =* 0.031).
de Oliveira et al. (52) Brazil Cross-sectional	Medical University Hospital of the Federal University of Uberlândia	TMJ Disorders: 125 Controls: 41	Mean age for TMJ disorders and controls: ≥18 years	Fonseca's questionnaire	Anamnesis Index and the State-Trait Anxiety Inventory (IDATE).	Chi-square tests *via* Monte Carlo simulation (*p* = 0.142)	Chi-square test indicates that TMD severity is independent of the trait anxiety. However, TMD severity is associated with the severity of state anxiety (*p* = 0.0410)
Yu et al. (53) China Cross-sectional	Pilots from Shenzhen Airlines	TMJ Disorders: 205 Controls: 411	Mean age for TMJ disorders and controls: 31.4 ± 5.9	Clinical examination contained TMD screening per Research Diagnostic Criteria for TMD (RDC/TMD).	Trait Anxiety section of Spielberger State-Trait Anxiety Inventory (STAI-T).	Kruskal–Wallis; Chi-square test and multiple logistic regression models *p* ≤ 0.05	The STAI-T score in TMD group was significantly higher compared with non-TMD. Thus, subjects with higher anxiety were more likely suffering from TMD.
Schmidt et al. (54) Brazil Case-control	Dental Clinic in the interior on the state of Rio Grande do Sul	TMJ Disorders: 20 Controls: 20	Mean age for TMJ disorders and controls: ≥18 years	TMD diagnosis performed through the specialized dental team	Young Schemes Questionnaire–reduced form (YSQ–S2), Beck Anxiety Questionnary (BAI):	Mann-Whitney e Wilcoxon *p* = 0.05	The statistics showed significant differences between the presence of more anxiety symptoms in the group with DTM, by applying the Mann-Whitney and Wilcoxon (*p =* 0.0191). Based on the findings of this study, it is clear that, despite being a maxillofacial pathology, TMD is inevitably associated with psychological factors such as anxiety.
Reissmann et al. (17) Germany Case-control	Departament of prosthodontics and materials science, University of Leipzig. Departament of prosthodontics, Martin Luther University Halle-	TMJ Disorders: 320 Controls: 888	Mean age for TMJ Disorders: 39.4 ± 15.4 Mean age for Controls: 40.4 ± 11.8	German version of Research Diagnostic Criteria for Temporomandibular Disorders (RDC/TMD)	State-Trait Anxiety Inventory (STAI)	Logistic regression analysis Students' *t-*test	Trait anxiety was more pronounced in TMD patients than in controls (*t* test: *p < * 0.001). The patient's trait anxiety was more often classified as moderate or severe. In the logistic regression analysis, a one-point increase in STAI-Trait summary scores resulted in 1.04–fold higher odds of having pain-related TMD (*p* < 0.001) compared to controls.
Sójka et al. (55) Poland Cross-sectional	Poznań University of Medical Sciences	TMJ Disorders: 90 Controls: 181	Mean age for TMJ disorders and controls: 21.28 (35–37, 39–42, 44, 45, 47–49, 53, 55, 56) years	Diagnostic Criteria for Temporomandibular disorders axis I (DC/TMD)	Four-Dimensional Questionnaire (4DSQ); Sense of coherence orientation to life questionnaire	Spearman rho correlation and Mann-Whitney *P < * 0.05	About one-third of the students in this study presented symptoms of TMD and perceived more intensively symptoms of anxiety.
Staniszewski et al. (56) Norway Case-control	National TMD project in Bergen, Norway	TMJ Disorders: 44 Controls: 44	Mean age for TMJ Disorders: 44 Mean age for Controls: 46	Investigation of the severity and duration of symptoms, both for pain and dysfunction, analyzing pain intensity and duration, and functional impairment (general and jaw-specific).	Hospital Anxiety and Depression Scale (HADS) and a 2-item version of the Coping Strategies Questionnaire.	Wilcoxon signed rank test; linear multiregression between; and linear correlation *P < * 0.05	Anxiety scores and pain catastrophizing scores were significantly higher in the TMD group compared to Controls.
Tay et al. (6) Singapore Cross-sectional	Military dental centers of Singapore Armed Forces (SAF).	TMJ Disorders: 742 Controls: 1,301	Mean age for TMJ disorders and controls: 24.18 ± 7.18 years	Research Diagnostic Criteria for TMD (RDC/TMD) Symptom Questionnaire (SQ) to assess pain characteristics, history of headaches, jaw joint noises as well as closed / open jaw locking in the past 30 days.	The Depression, Anxiety and Stress Scale-21 (DASS-21).	Spearman correlation test *p < * 0.05	Specific type and number of TMD symptoms impacted OHRQoL and psychological states differently. Associations between number of TMD symptoms and quality of life, depression, anxiety and stress were significant but weak.
Bastos et al. (5) Brazil Case-control	Department of Dentistry of the Federal University of Rio Grande do Norte (UFRN), Natal, Brazil	TMJ Disorders: 60 Controls: 60	Mean age for TMJ disorders and controls: 33.29 ± 13.68 years	Research Diagnostic Criteria for Temporomandibular Disorders (RDC/TMD)	Anxiety (Beck Anxiety Inventory [BAI]; the State-Trait Anxiety Inventory [STAI-S and -T]; the Hospital Anxiety and Depression Scale [HADS])	X^2^ test Student *t* test Odds ratio (OR) analysis Non-conditional logistic regression	Anxiety was observed by a higher percentage of TMD participants (75 percent; *p* < 0.001) in the HADS test. According to STAI-S and -T, the majority of TMD patients experienced anxiety (55.6%). In terms of BAI, the majority of anxious people had TMDs (63.9%).
do Patrocinio et al. (7) Brazil Cross-sectional	Dental students at the Federal University of Campina Grande, Patos, Brazil.	TMJ Disorders: 144 Controls: 41	Mean age for TMJ disorders and controls: 21.4 (18–38) years	Fonseca's questionnaire	The State-Trait Anxiety Inventory [STAI-S and -T];	Chi-square Fisher exact *P* = 0.05	Statistical analysis revealed that the presence or absence of TMJ dysfunction had no effect on state (*p =* 0.297) or trait anxiety (*p =* 0.484). In terms of anxiety-related outcomes, it was discovered that most undergraduates had moderate state-anxiety based on the results of the State-Trait Anxiety Inventory - STAI.
Nguyen et al. (16) Vietnam Cross-sectional	Participants were randomly selected based on sex and residence living in Danang, Vietnam.	TMJ Disorders:75 Controls: 104	Mean age for TMJ disorders and controls: 65–74 years	Orthopantomography set at 73 kV, 10 mA, and 17.6 s with a CC-detector sensor (Soredex Cranex D, Tuusula, Finland).	Self-reported 7-item Generalized Anxiety Disorder Scale (GAD-7)	SPSS Kruskal-Wallis test Spearman's test *P < * 0.05	Positive correlations were found between limitation of mandibular function and anxiety (r = 0.304, *p < * 0.001). TMJ osseous changes were not correlated with anxiety.

The included studies were carried out in 16 countries: Brazil (*n* = 10), Canada (*n* = 2), China (*n* = 2), Croatia (*n* = 1), Germany (*n* = 3), India (*n* = 1), Italy (*n* = 1), Nigeria (*n* = 1), Norway (*n* = 1), Poland (*n* = 1), Scotland (*n* = 1), Vietnam (*n* = 1), Singapore (*n* = 1), Socialist Democratic Republic of Sri Lanka (*n* = 1), Thailand (*n* = 1), and USA (*n* = 5).

Therefore, it is clear that the results used in this review came from epidemiological studies that used samples from 4 of the 5 continents of the globe.

In terms of sample origin, 14 studies used patients from dental schools or specific university departments in the area; 14 studies used patients from hospitals and/or large dental health centers; 5 from a university population; 5 from the general community selected according to the authors' methods; 3 from private dental offices; and 2 from university institutes. Certain studies may have developed their samples from more than one of the locations mentioned above.

The Research Diagnostic Criteria for Temporomandibular Disorders (RDC/TMD) (axis I and/or II) was the most commonly used parameter for the evaluation of TMD, followed by Fonseca's Anamnestic Index (FAI) and the Academy of Orofacial Pain's Guidelines for Diagnosis of TMD, besides the clinical evaluation of the signs and symptoms presented. For anxiety assessment, several instruments were available: Minnesota Multiphasic Personality Inventory (MMPI), Speilberger State-Trait Anxiety Inventory (STAI), The State-Trait Anxiety Inventory [STAI-S and -T]; Eysenck Personality Questionnaire (EPQ), Pennybaker Inventory of Limbic Languidness (PILL), Scale ranging from 1 to 5 (from very relaxed to extremely tense) after the electromyographical evaluation, Hospital Anxiety and Depression Scale questions (HADS), The Crown Crisp Experimental Index (CCEI), Taylor Manifest Anxiety State, State-Trait Personality Inventory (STPI), Emotion Assessment Scale (EAS), Positive and Negative Affect Scale (PANAS), Visual Analog Scale (VAS), The Symptom Checklist 90—Revised (SCL-90R); SCL-90 Questionnaire, The symptom check list of 90 revised questionnaire (SCL-90R), Self-report questionnaire PAS-SR (version of SCIPAS), The 28-item General Health Questionnaire (GHQ-28), Beck Anxiety Inventory (BAI), Life Events Scale, Anamnesis Index and the State-Trait Anxiety Inventory (IDATE), Composite International diagnostic Screener (CID-S), Zung self-rating scale, Young Schemes Questionnaire—reduced form, Young Schemes Questionnaire—reduced form, Four Dimensional Questionnaire (4DSQ), Sense of Coherence Orientation to Life Questionnaire, 2-item version of the Coping Strategies Questionnaire, The Depression, Anxiety and Stress Scale-21 (DASS-21), and Self-reported 7-item Generalized Anxiety Disorder Scale (GAD-7).

### Qualitative synthesis of the studies

Tables 2, 3 summarize the findings of the NOS methodological quality assessment. As a result, 15 studies were judged to be of high quality (6–8, 16, 28, 34, 39, 40, 45, 47–49, 53, 55, 56); 10 as medium quality (35–37, 41, 42, 44, 50, 51, 54); and 9 as low quality with a high risk of bias (5, 17, 29–31, 38, 46, 52, 58).

**Table 2:** Newcastle-Ottawa Scale for cross-sectional studies.

**Studies**		**Xu et al. (43)**	**Fernandes et al. (44)**	**Monteiro et al. (47)**	**Bezerra et al. (48)**	**Boscato et al. (49)**	**Smriti et al. (50)**	**Lemos et al. (51)**	**Oliveira et al. (52))**	**Yu et al. (53)**	**Sójka et al. (55)**	**do Patrocinio et al. (7)**	**Nguyen et al. (16)**	**Tay et al. (6)**
Selection	Representativeness of the sample	*	–	*	*	*	*	*	–	*	*	*	*	*
	Sample size	–	–	*	*	*	–	–	–	–	–	*	*	*
	Non–respondents	–	–	*	*	*	–	–	–	*	*	–	*	*
	Ascertainment of the exposure	**	**	**	**	**	**	**	**	**	**	**	**	**
Comparability		–	**	**	**	**	*	*	–	**	*	*	**	**
Outcome	Assessment of the outcome	*	*	*	*	*	*	*	*	*	*	*	*	*
	Statistical test	*	*	*	–	*	*	*	*	*	*	*	*	*
Quality		POOR	FAIR	GOOD	GOOD	GOOD	FAIR	FAIR	POOR	GOOD	GOOD	GOOD	GOOD	GOOD

The number of ^*^ symbol indicates the number of points assigned to a given parameter.

**Table 3:** Newcastle-Ottawa Scale for case-control studies.

**Studies**		**Solberg et al. (28)**	**Moss and Adams (29)**	**Southwell et al. (30)**	**Schroeder et al. (31)**	**Stockstill and Callahan (35)**	**Zach and Andreasen (57)**	**Curran et al. (36)**	**Jones et al. (37)**	**Sirirungrojying et al. (38)**	**Velly et al. (39)**	**Manfredini et al. (40)**	**Pallegama et al. (41)**	**Saheeb and Otakpor (42)**	**Vedolin et al. (45)**	**Giannakopoulos et al. (8)**	**Lajnert et al. (46)**	**Schmidt et al. (54)**	**Reissmann et al. (17)**	**Staniszewski et al. (56)**	**Bastos et al. (5)**
Selection	Case definition adequate	*	–	–	*	–	*	*	*	*	*	*	*	*	*	*	*	–	*	*	*
	Representativeness of the cases	–	–	–	–	–	*	–	*	–	–	–	–	–	*	–	–	–	*	*	–
	Selection of controls	*	*	*	–	*	*	*	*	*	*	–	*	*	*	*	–	*	–	*	*
	Definition of controls	*	*	–	–	*	*	–	–	–	*	*	*	*	*	*	–	*	–	*	–
Comparability		*	*	*	*	**	*	**	**	*	**	**	**	**	*	*	*	*	*	**	*
Exposure	Ascertainment of exposure	*	–	–	–	–	–	*	–	–	*	*	–	–	*	–	–	–	–	*	–
	Same method of ascertainment for cases and controls	*	*	*	*	*	*	*	*	*	*	*	*	*	*	*	*	*	*	*	*
	Non–Response rate	–	–	–	–	*	*	–	–	–	*	–	–	–	–	*	–	*	*	*	–
Quality		GOOD	POOR	POOR	POOR	FAIR	GOOD	FAIR	FAIR	GOOD	POOR	GOOD	FAIR	FAIR	GOOD	GOOD	POOR	FAIR	POOR	GOOD	POOR

The number of * symbol indicates the number of points assigned to a given parameter.

The main issues found in studies with a high risk of bias were case representativeness, no control group definition, lack of information about the verification of exposure, and no description of the non-response rate. Minimum defects in the representativeness of the cases, sample size, and non-response rate were perceived in articles with a moderate risk of bias. In studies that demonstrated a low risk of bias, only the verification of the exposure offered a minimum issue, since the assessment of anxiety was performed using questionnaires. Hence, according to the qualifier, questionnaires are regarded as a probable source of bias.

### Quantitative analysis (meta-analysis)

We could not conduct a meta-analysis due to differences in study methodologies, such as subject age, TMD diagnosis, and finally, although the authors used the same anxiety indices (such as HADS and STAI-T), the outcomes were presented in different ways (median, mean, frequency, etc.). Furthermore, studies with the same TMD diagnosis and anxiety analysis methodology, as well as participants of similar ages, had varying methodological quality. As a result, the meta-results analysis's would be inconsistent and highly heterogeneous. Therefore, we decided to only include a summary of findings from the GRADE system.

### Level of evidence (GRADE)

The level of evidence was evaluated for the following outcomes: anxiety-trait and TMD are not related; anxiety-state and TMD are positively related; anxiety-trait and state and TMD are positively related; anxiety-symptoms and TMD are positively related; anxiety-level and TMD are positively related. In general, the certainty of the evidence was rated as low and very low. Many of the studies included in the syntheses presented methodological limitations that could have seriously affected the estimates reported. Clearly, the methodological heterogeneity among the studies explains the inconsistency of the results. Finally, even for the outcomes that included more studies, the number of individuals considered when synthesizing the information was small; thus, it was deemed that the imprecision item was seriously affected. Only the outcome “Anxiety symptoms and TMD are positively related” did not demonstrate any serious problem, thus showing a low certainty of evidence. The results of certainty of evidence results are described in Table 4.

**Table 4:** Certainty of evidence.

**Certainty assessment**							**No. of patients**		**Certainty**
**No. of studies**	**Study design**	**Risk of bias**	**Inconsistency**	**Indirectness**	**Imprecision**	**Other considerations**	**With TMJ**	**Controls**	
**Anxiety - Trait and TMD are not related**.									
1	Observational studies	Serious^a^	Not serious	Not serious	Serious^b^	None	47/-	49/-	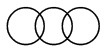 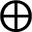 Very low
**Anxiety - State and TMD are positively related**.									
14	Observational studies	Serious^c^	Serious^d^	Not serious	Not serious	None	1,252/-	2,328/-	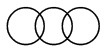 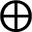 Very low
**Anxiety - Trait and State and TMD are positively related**.									
13	Observational studies	Serious^e^	Serious^f^	Not serious	Not serious	None	1,475/-	1,873/-	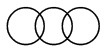 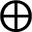 Very low
**Anxiety- symptoms and TMD are positively related**.									
3	Observational studies	Not serious	Not serious	Not serious	Not serious	None	852/-	1,374/-	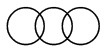 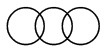 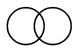 Low
**Anxiety - disorder**									
2	Observational studies	Not serious	Not serious	Not serious	Not serious	None	179/-	285/-	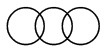 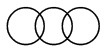 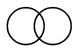 Low
**Anxiety - Level and TMD are positively related**.									
1	Observational studies	Serious^a^	Not serious	Not serious	Serious ^b^	None	28/-	122/-	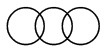 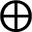 Very low

CI, confidence interval. a. All included studies presented some type of risk of bias. b. Total number of participants is less than 400. c. Only 43% of included studies was classified as “good” on risk of bias assessment. d. 14 studies reported that anxiety and TMD are positive related, while 2 studies reported no difference between groups. e. Only 30.8% of included studies was classified as “good” on risk of bias assessment. f. 13 studies reported that anxiety and TMD are positive related, while 2 studies reported no difference between groups.

## Discussion

The relationship between TMD and psychosocial impairment is widely explored in the literature (12–14, 56). However, despite the specific association between TMD and anxiety has been the subject of some studies (5, 8–11), to our knowledge, this is the first systematic review that has evaluated this relationship individually. In the quantitative analysis, our results confirmed the association between anxiety and DTM, although a low certainty of evidence among the selected studies.

Several assessment instruments were used for the classification of TMD. It was noted that before the elaboration of the RDC/TMD (59), with took place in 1992, all studies (28–30, 34, 35) used different instruments, which were mainly based on clinical story, signs and symptoms, clinical diagnosis of TMJ and masticatory muscles. Only one study (31) used a validated questionnaire, The Helkimo Clinical Dysfunction Index (HCDI). Half of these studies indicated that patients with TMD showed higher state and/or trait anxiety levels, while the other half did not show significant differences regarding anxiety or anxiety traits between patients with and without TMD. The lack of standardization in the diagnosis of TMD may have influenced the studies' findings and made it impossible to compare them.

Since the introduction of the RDC/TMD (59), this has been used especially to standardize research findings and represented an important advance as it is composed of two axes: Axis I, with an emphasis on physical diagnosis, and Axis II, addresses biopsychosocial aspects and pain-related disability. From the emergence of this assessment instrument, evidence demonstrating the importance of psychological assessment and pain disability has been growing and psychological factors came to be considered at least as important for treatment outcome as pain intensity and physical diagnoses (60). However, not all selected studies have used RDC/TMD. Only 13 did so (5, 6, 8, 17, 36, 37, 39, 40, 42, 45–47, 53); while another 14 used different assessment methods, such as Fonseca Anamnestic Index and Diagnostic Criteria of The American Academy of Craniomandibular Disorders (7, 16, 38, 41, 43, 44, 48–52, 54, 56, 61).

Among the studies that used the RDC/TMD, 11 obtained results that were positively associated TMD with anxiety. Only 3 mentioned the related TMD subtype, such as myofascial pain or joint pain (39, 40, 42). A previous study (62) evaluated the correlation between Axis I and Axis II diagnosis of the RDC/TMD and investigated whether the presence of pain could mediate correlation to them. The authors concluded that there is no specific correlation between the two Axes findings and identified that the presence of pain, whether of muscular or joint origin, was correlated with Axis II findings. Besides that higher levels of pain-related impairment were associated with the most severe score of psychological symptoms evaluated, depression and somatization.

Among the studies evaluated in the present study, only one (55) has used DC/TMD (63), a more recent and updated RDC/TMD version, with the purpose of both research and clinical use. This assessment instrument includes revised and new validated tools for both the physical diagnosis (Axis I) and the psychological and disability assessment (Axis II). The latter Axis offers a brief assessment with a minimal number of short screening instruments for the most important variables that may influence the development or perpetuation of symptoms, or an expanded assessment with a more comprehensive set of instruments, some specific to orofacial pain (60). Perhaps just only this study has used it, because this is a relatively new tool, and the translation and validation of this questionnaire for other languages took place gradually (2). In the specific case of this study, the results demonstrated that one-third of the evaluated individuals presented symptoms of TMD and perceived more intensive symptoms of anxiety.

In none of the articles included in our systematic review, the International Classification of Orofacial Pain (ICOP) was used, most likely because it is a very recent instrument (64).

Regarding the diagnosis of anxiety, several validated tools were used. Among these, the most used were the following: The Spielberger State-Trait Anxiety Inventory (STAI) (65), which was used as the only assessment instrument by 7 studies (7, 17, 29, 41, 44, 47, 53). It consists of 2 self-administered questionnaires: one, about State-anxiety (STAI-S), a transient emotional state or condition that is characterized by consciously perceived unpleasant feelings of tension and increased activity of the autonomic nervous system; and the other, about Trait-anxiety (STAI-T), relatively stable individual differences in anxiety propensity, the difference in reacting into situations perceived as threatening, which can increase the intensity of anxiety state (66). Another tool was the Hospital Anxiety and Depression Scale (HADS), a reliable instrument for screening clinically significant anxiety and depression (33). It was used by 3 studies (8, 49, 51). Also, The Symptom Checklist 90 (SCL-90 Questionnaire), both in its revised and complete version (38, 39, 43) and comprises a 90-item self-report symptom scale, multidimensional, which included subcategories of somatization, obsessive-compulsive symptoms, interpersonal sensitivity, depression, anxiety, hostility, phobic anxiety, paranoid ideation, and psychoticism (67). In fact, some studies used more than one assessment instrument (5, 30, 31, 36, 37, 42, 46), and although this difference between methodological protocols makes it difficult to compare the results of the analyzed studies, it is possible to observe that most of them obtained as results a positive association between TMD and anxiety, i.e., patients with TMD had higher levels of anxiety when compared to individuals without TMD.

In summary, although all the articles selected in our systematic review have very different methodological approaches, with very diverse samples as well. Therefore some aspects are found to be coinciding with those previously described in the literature. For example, in most studies, the prevalence of TMD, as well as anxiety, was higher in women who were in adulthood, in an age group between 20 and 40 years. In these patients, the main signs and symptoms of TMD were muscle and/or joint pain and limitation in mandibular range of motion. All of these lasted more than 6 months and, in some cases, even years.

Previous systematic reviews have already revealed this positive association between TMD and psychosocial factors. Recently, Reis et al. (18) demonstrated the relationship between distinct subtypes of TMD and anxiety and depression. These results suggest that patients with myofascial pain are more anxious and more depressed than patients with other subtypes of TMD, especially disc displacement or arthralgia/degenerative joint disease. The authors suggest that the lower sensitivity of the RDC/TMD in arthralgia diagnosis could have overestimated myofascial pain diagnosis. Häggman-Henrikson et al. (12) suggest an association between catastrophizing and TMD. The findings of these authors pointed not only a higher level of catastrophizing in TMD patients but also an association between levels of catastrophizing, symptoms severity, and treatment outcomes. For the authors, higher levels of catastrophizing before treatment can be associated with patients who do not respond to treatment and report higher activity interference 1 year later. De La Torre Canales et al. (14) indicate that psychological disorders and psychosocial impairment are highly prevalent in TMD patients, mainly severe-to-moderate somatization and depression. Severe physical impairment was not commonly reported in this study.

Some hypotheses were elaborated by the studies selected in the present systematic review to explain this association between TMD and anxiety. Boscato et al. (49) emphasized that anxiety plays an important role in TMD and can be considered as an initiating or aggravating factor. For them, during clinical evaluation, anxious individuals report greater pain intensity. de Oliveira et al. (52) highlighted that trait anxiety appeared to play a role mainly as an etiological agent, whereas the anxiety state had a more psychosomatic impact on TMD severity. Monteiro et al. (47) ratified two basic concepts cited in the literature to explain this association: individuals with neuroticism tend to be often anxious and anxious subjects could increase attention to pain, thereby amplifying their perceived intensity. For Reissmann et al. (68), anxiety has an important role in TMD and, in this way, a person's general disposition to be anxious can be considered as a risk factor for TMD pain. From the results found in their study, Staniszewski et al. (56) hypothesized that psychological factors may contribute to chronic upregulation of the HPA axis, with higher salivary cortisol (*F*) secretion from the adrenal cortex.

The findings of the present systematic review are specific to TMD and anxiety. The level of evidence evaluation thought GRADE assessment presents a low level, which is related to observational studies evaluated since methodological discrepancies, such as different studies design, representativeness of the sample, sample size, control group criteria, and non-valid diagnostics instruments. Despite the low certainty of the evidence, it is important to note that the most selected studies demonstrated the association between TMD and anxiety.

## Conclusions

Considering the limitations of this systematic review, it can be suggested a significant association between anxiety and TMD, as well as highlights possible directions for future research. Thereby, to establish a high certainty of evidence related to the association between anxiety and TMD, it is necessary to carry out further studies that focus on one of the TMD subtypes (articular or muscular) and that assess anxiety as a risk factor for the initiation, development, or perpetuation of TMD, which can make it difficult for patients to respond to TMD treatment.

## Data availability statement

The original contributions presented in the study are included in the article/Supplementary material, further inquiries can be directed to the corresponding author/s.

## Author contributions

ES, BP, and RS-R: study concept and design. ES, BP, DF, YN, NF, MM, LM, RRL, and RS-R: analysis and interpretation of data. ES, BP, DF, and YN: preparation of the manuscript. NF, MM, LM, RRL, and RS-R: critical revision of the manuscript. All authors contributed to the article and approved the submitted version.

## Funding

RRL is a researcher from Conselho Nacional de Desenvolvimento Científico e Tecnológico (CNPq) and received Grant No. 312275/2021-8. The Article Processing Charges (APC) was funded by Pró-Reitoria de Pesquisa e Pós-graduação from Federal University of Pará (PROPESP-UFPA).

## Conflict of interest

The authors declare that the research was conducted in the absence of any commercial or financial relationships that could be construed as a potential conflict of interest.

## Publisher's note

All claims expressed in this article are solely those of the authors and do not necessarily represent those of their affiliated organizations, or those of the publisher, the editors and the reviewers. Any product that may be evaluated in this article, or claim that may be made by its manufacturer, is not guaranteed or endorsed by the publisher.
