# Supplementary material for: Association between temporomandibular disorders and anxiety: A systematic review
Source: Front Psychiatry. 2022 Oct 13;13:990430. doi: 10.3389/fpsyt.2022.990430 (PMC9606663; doi:10.3389/fpsyt.2022.990430)
Supplement: Supplementary file 1 [file Table_1.DOCX]

Supplementary table 1: Terms used on databases searches.

| Database | Search format |
| --- | --- |
| PUBMED | ((((((((((Humans[MeSH Terms]) OR Humans[Title/Abstract]) OR Homo sapiens[Title/Abstract]) OR Man AND (Taxonomy) AND Title/Abstract) OR Man, Modern[Title/Abstract]) OR Modern Man[Title/Abstract]) OR Human[Title/Abstract])) AND ((((((Adult[MeSH Terms]) OR Adult[Title/Abstract]) OR Adults[Title/Abstract]) OR Aged[MeSH Terms]) OR Aged[Title/Abstract]) OR Elderly[Title/Abstract])) AND ((((((((((((((((((((((((((((((((((((((((((((((((((((temporomandibular joint disorders[MeSH Terms]) OR Temporomandibular Joint Disorders[Title/Abstract]) OR Disorder, Temporomandibular Joint[Title/Abstract]) OR Disorders, Temporomandibular Joint[Title/Abstract]) OR Joint Disorder, Temporomandibular[Title/Abstract]) OR Joint Disorders, Temporomandibular[Title/Abstract]) OR Temporomandibular Joint Disorder[Title/Abstract]) OR TMJ Disorders[Title/Abstract]) OR Disorder, TMJ[Title/Abstract]) OR Disorders, TMJ[Title/Abstract]) OR TMJ Disorder[Title/Abstract]) OR Temporomandibular Disorders[Title/Abstract]) OR Disorder, Temporomandibular[Title/Abstract]) OR Disorders, Temporomandibular[Title/Abstract]) OR Temporomandibular Disorder[Title/Abstract]) OR Temporomandibular Joint Diseases[Title/Abstract]) OR Disease, Temporomandibular Joint[Title/Abstract]) OR Diseases, Temporomandibular Joint[Title/Abstract]) OR Joint Disease, Temporomandibular[Title/Abstract]) OR Joint Diseases, Temporomandibular[Title/Abstract]) OR Temporomandibular Joint Disease[Title/Abstract]) OR TMJ Diseases[Title/Abstract]) OR Disease, TMJ[Title/Abstract]) OR Diseases, TMJ[Title/Abstract]) OR TMJ Disease[Title/Abstract]) OR Temporomandibular Joint[MeSH Terms]) OR Temporomandibular Joint[Title/Abstract]) OR Joint, Temporomandibular[Title/Abstract]) OR Joints, Temporomandibular[Title/Abstract]) OR Temporomandibular Joints[Title/Abstract]) OR TMJ[Title/Abstract]) OR temporomandibular joint dysfunction syndrome[MeSH Terms]) OR Temporomandibular Joint Dysfunction Syndrome[Title/Abstract]) OR Myofascial Pain Dysfunction Syndrome, Temporomandibular Joint[Title/Abstract]) OR TMJ Syndrome[Title/Abstract]) OR Syndrome, TMJ[Title/Abstract]) OR Costen's Syndrome[Title/Abstract]) OR Costen Syndrome[Title/Abstract]) OR Costens Syndrome[Title/Abstract]) OR Syndrome, Costen's[Title/Abstract]) OR Temporomandibular Joint Syndrome[Title/Abstract]) OR Joint Syndrome, Temporomandibular[Title/Abstract]) OR Syndrome, Temporomandibular Joint[Title/Abstract]) OR myofascial pain syndromes[MeSH Terms]) OR Myofascial Pain Syndromes[Title/Abstract]) OR Myofascial Pain Syndrome[Title/Abstract]) OR Pain Syndrome, Myofascial[Title/Abstract]) OR Pain Syndromes, Myofascial[Title/Abstract]) OR Syndrome, Myofascial Pain[Title/Abstract]) OR Syndromes, Myofascial Pain[Title/Abstract]) OR Myofascial Trigger Point Pain[Title/Abstract]) OR Trigger Point Pain, Myofascial[Title/Abstract])) AND ((((((((((((((((((((((Anxiety[MeSH Terms]) OR Anxiety[Title/Abstract]) OR Hypervigilance[Title/Abstract]) OR Nervousness[Title/Abstract]) OR Social Anxiety[Title/Abstract]) OR Anxieties, Social[Title/Abstract]) OR Anxiety, Social[Title/Abstract]) OR Social Anxieties[Title/Abstract]) OR Anxious[Title/Abstract]) OR anxiety disorders[MeSH Terms]) OR Anxiety Disorders[Title/Abstract]) OR Anxiety Disorder[Title/Abstract]) OR Disorder, Anxiety[Title/Abstract]) OR Disorders, Anxiety[Title/Abstract]) OR Neuroses, Anxiety[Title/Abstract]) OR Anxiety Neuroses[Title/Abstract]) OR Anxiety States, Neurotic[Title/Abstract]) OR Anxiety State, Neurotic[Title/Abstract]) OR Neurotic Anxiety State[Title/Abstract]) OR Neurotic Anxiety States[Title/Abstract]) OR State, Neurotic Anxiety[Title/Abstract]) OR States, Neurotic Anxiety[Title/Abstract]) |
| SCOPUS | ( TITLE-ABS-KEY ( human* ) OR TITLE-ABS-KEY ( "modern man" ) OR TITLE-ABS-KEY ( "man (taxonomy)" ) OR TITLE-ABS-KEY ( "homo sapiens" ) AND TITLE-ABS-KEY ( adult* ) OR TITLE-ABS-KEY ( aged ) OR TITLE-ABS-KEY ( elderly ) ) AND ( TITLE-ABS-KEY ( "temporomandibular joint disorder*" ) OR TITLE-ABS-KEY ( "tmj disorder*" ) OR TITLE-ABS-KEY ( "temporomandibular disorder*" ) OR TITLE-ABS-KEY ( "temporomandibular joint disease*" ) OR TITLE-ABS-KEY ( "tmj disease*" ) OR TITLE-ABS-KEY ( "temporomandibular joint*" ) OR TITLE-ABS-KEY ( "tmj" ) OR TITLE-ABS-KEY ( "temporomandibular joint dysfunction syndrome" ) OR TITLE-ABS-KEY ( "myofascial pain dysfunction syndrome, temporomandibular joint" ) OR TITLE-ABS-KEY ( "tmj syndrome" ) OR TITLE-ABS-KEY ( "costen's syndrome" ) OR TITLE-ABS-KEY ( "costen* syndrome" ) OR TITLE-ABS-KEY ( "temporomandibular joint syndrome" ) OR TITLE-ABS-KEY ( "myofascial pain syndrome*" ) OR TITLE-ABS-KEY ( "myofascial trigger point pain" ) ) AND ( TITLE-ABS-KEY ( anxiety ) OR TITLE-ABS-KEY ( hypervigilance ) OR TITLE-ABS-KEY ( nervousness ) OR TITLE-ABS-KEY ( "social anxiet*" ) OR TITLE-ABS-KEY ( anxious ) OR TITLE-ABS-KEY ( "anxiety disorder*" ) OR TITLE-ABS-KEY ( "anxiety neuroses" ) OR TITLE-ABS-KEY ( "neurotic anxiety state*" ) ) |
| COCHRANE | #1: human* OR "Modern Man” OR "Man (Taxonomy)” OR "Homo sapiens"  #2: “Adult” OR “Adults” OR “Aged” OR “Elderly”  #3: #1 AND #2  #4: “Temporomandibular Joint Disorders” OR “Disorder, Temporomandibular Joint” OR “Disorders, Temporomandibular Joint” OR “Joint Disorder, Temporomandibular” OR “Joint Disorders, Temporomandibular” OR “Temporomandibular Joint Disorder” OR “TMJ Disorders” OR “Disorder, TMJ” OR “Disorders, TMJ” OR “TMJ Disorder” OR “Temporomandibular Disorders” OR “Disorder, Temporomandibular” OR “Disorders, Temporomandibular” OR “Temporomandibular Disorder” OR “Temporomandibular Joint Diseases” OR “Disease, Temporomandibular Joint” OR “Diseases, Temporomandibular Joint” OR “Joint Disease, Temporomandibular” OR “Joint Diseases, Temporomandibular” OR “Temporomandibular Joint Disease” OR “TMJ Diseases” OR “Disease, TMJ” OR “Diseases, TMJ” OR “TMJ Disease” OR “Temporomandibular Joint” OR “Joint, Temporomandibular” OR “Joints, Temporomandibular” OR “Temporomandibular Joints” OR “TMJ” OR “Temporomandibular Joint Dysfunction Syndrome” OR “Myofascial Pain Dysfunction Syndrome, Temporomandibular Joint” OR “TMJ Syndrome” OR “Syndrome, TMJ” OR “Costen* Syndrome” OR “Costen Syndrome” OR “Costens Syndrome” OR “Syndrome, Costen's” OR “Temporomandibular Joint Syndrome” OR “Joint Syndrome, Temporomandibular” OR “Syndrome, Temporomandibular Joint” OR “Myofascial Pain Syndromes” OR “Myofascial Pain Syndrome” OR “Pain Syndrome, Myofascial” OR “Pain Syndromes, Myofascial” OR “Syndrome, Myofascial Pain” OR “Syndromes, Myofascial Pain” OR “Myofascial Trigger Point Pain” OR “Trigger Point Pain, Myofascial”  #5: “Anxiety” OR “Hypervigilance” OR “Nervousness” OR “Social Anxiety” OR “Anxieties, Social” OR “Anxiety, Social” OR “Social Anxieties” OR “Anxious” OR “anxiety disorders” OR “anxiety disorder” OR “Disorder, Anxiety” OR “Disorders, Anxiety” OR “Neuroses, Anxiety” OR “Anxiety Neuroses” OR “Anxiety States, Neurotic” OR “Anxiety State, Neurotic” OR “Neurotic Anxiety State” OR “Neurotic Anxiety States” OR “State, Neurotic Anxiety” OR “States, Neurotic Anxiety”  #6: #3 AND #4 AND #5 |
| WEB OF SCIENCE | TS=( human* OR "Modern Man” OR "Man (Taxonomy)” OR "Homo sapiens") AND TS=(“Adult” OR “Adults” OR “Aged” OR “Elderly”) AND TS“Temporomandibular Joint Disorders” OR “Disorder, Temporomandibular Joint” OR “Disorders, Temporomandibular Joint” OR “Joint Disorder, Temporomandibular” OR “Joint Disorders, Temporomandibular” OR “Temporomandibular Joint Disorder” OR “TMJ Disorders” OR “Disorder, TMJ” OR “Disorders, TMJ” OR “TMJ Disorder” OR “Temporomandibular Disorders” OR “Disorder, Temporomandibular” OR “Disorders, Temporomandibular” OR “Temporomandibular Disorder” OR “Temporomandibular Joint Diseases” OR “Disease, Temporomandibular Joint” OR “Diseases, Temporomandibular Joint” OR “Joint Disease, Temporomandibular” OR “Joint Diseases, Temporomandibular” OR “Temporomandibular Joint Disease” OR “TMJ Diseases” OR “Disease, TMJ” OR “Diseases, TMJ” OR “TMJ Disease” OR “Temporomandibular Joint” OR “Joint, Temporomandibular” OR “Joints, Temporomandibular” OR “Temporomandibular Joints” OR “TMJ” OR “Temporomandibular Joint Dysfunction Syndrome” OR “Myofascial Pain Dysfunction Syndrome, Temporomandibular Joint” OR “TMJ Syndrome” OR “Syndrome, TMJ” OR “Costen* Syndrome” OR “Costen Syndrome” OR “Costens Syndrome” OR “Syndrome, Costen's” OR “Temporomandibular Joint Syndrome” OR “Joint Syndrome, Temporomandibular” OR “Syndrome, Temporomandibular Joint” OR “Myofascial Pain Syndromes” OR “Myofascial Pain Syndrome” OR “Pain Syndrome, Myofascial” OR “Pain Syndromes, Myofascial” OR “Syndrome, Myofascial Pain” OR “Syndromes, Myofascial Pain” OR “Myofascial Trigger Point Pain” OR “Trigger Point Pain, Myofascial”) AND TS=(“Anxiety” OR “Hypervigilance” OR “Nervousness” OR “Social Anxiety” OR “Anxieties, Social” OR “Anxiety, Social” OR “Social Anxieties” OR “Anxious” OR “anxiety disorders” OR “anxiety disorder” OR “Disorder, Anxiety” OR “Disorders, Anxiety” OR “Neuroses, Anxiety” OR “Anxiety Neuroses” OR “Anxiety States, Neurotic” OR “Anxiety State, Neurotic” OR “Neurotic Anxiety State” OR “Neurotic Anxiety States” OR “State, Neurotic Anxiety” OR “States, Neurotic Anxiety”) |
| LILACS | human* OR "Modern Man” OR "Man (Taxonomy)” OR "Homo sapiens" AND “Adult” OR “Adults” OR “Aged” OR “Elderly” AND “Temporomandibular Joint Disorders” OR “Disorder, Temporomandibular Joint” OR “Disorders, Temporomandibular Joint” OR “Joint Disorder, Temporomandibular” OR “Joint Disorders, Temporomandibular” OR “Temporomandibular Joint Disorder” OR “TMJ Disorders” OR “Disorder, TMJ” OR “Disorders, TMJ” OR “TMJ Disorder” OR “Temporomandibular Disorders” OR “Disorder, Temporomandibular” OR “Disorders, Temporomandibular” OR “Temporomandibular Disorder” OR “Temporomandibular Joint Diseases” OR “Disease, Temporomandibular Joint” OR “Diseases, Temporomandibular Joint” OR “Joint Disease, Temporomandibular” OR “Joint Diseases, Temporomandibular” OR “Temporomandibular Joint Disease” OR “TMJ Diseases” OR “Disease, TMJ” OR “Diseases, TMJ” OR “TMJ Disease” OR “Temporomandibular Joint” OR “Joint, Temporomandibular” OR “Joints, Temporomandibular” OR “Temporomandibular Joints” OR “TMJ” OR “Temporomandibular Joint Dysfunction Syndrome” OR “Myofascial Pain Dysfunction Syndrome, Temporomandibular Joint” OR “TMJ Syndrome” OR “Syndrome, TMJ” OR “Costen* Syndrome” OR “Costen Syndrome” OR “Costens Syndrome” OR “Syndrome, Costen's” OR “Temporomandibular Joint Syndrome” OR “Joint Syndrome, Temporomandibular” OR “Syndrome, Temporomandibular Joint” OR “Myofascial Pain Syndromes” OR “Myofascial Pain Syndrome” OR “Pain Syndrome, Myofascial” OR “Pain Syndromes, Myofascial” OR “Syndrome, Myofascial Pain” OR “Syndromes, Myofascial Pain” OR “Myofascial Trigger Point Pain” OR “Trigger Point Pain, Myofascial” AND “Anxiety” OR “Hypervigilance” OR “Nervousness” OR “Social Anxiety” OR “Anxieties, Social” OR “Anxiety, Social” OR “Social Anxieties” OR “Anxious” OR “anxiety disorders” OR “anxiety disorder” OR “Disorder, Anxiety” OR “Disorders, Anxiety” OR “Neuroses, Anxiety” OR “Anxiety Neuroses” OR “Anxiety States, Neurotic” OR “Anxiety State, Neurotic” OR “Neurotic Anxiety State” OR “Neurotic Anxiety States” OR “State, Neurotic Anxiety” OR “States, Neurotic Anxiety” |
| GOOGLE SCHOLAR | "Temporomandibular joint disorders " + "Anxiety" -"in vitro" -"systematic review" -book |
| OPEN GREY | “Temporomandibular joint disorder” AND Anxiety |
